# Platelet-Mediated Transfer of Cardioprotection by Remote Ischemic Conditioning and Its Abrogation by Aspirin But Not by Ticagrelor

**DOI:** 10.1007/s10557-022-07345-9

**Published:** 2022-05-21

**Authors:** Helmut Raphael Lieder, Maria Tsoumani, Ioanna Andreadou, Karsten Schrör, Gerd Heusch, Petra Kleinbongard

**Affiliations:** 1 Institute for Pathophysiology, West German Heart and Vascular Centre, University of Essen Medical School, Essen, Germany; 2 Laboratory of Pharmacology, National and Kapodistrian University of Athens, Athens, Greece; 3 Department of Pharmacology and Clinical Pharmacology, Heinrich-Heine-University Düsseldorf, Düsseldorf, Germany

**Keywords:** Aspirin, Cardioprotection, Ischemia/reperfusion, Remote ischemic conditioning, Ticagrelor

## Abstract

**Purpose:**

The role of platelets during myocardial ischemia/reperfusion (I/R) is ambivalent. They contribute to injury but also to cardioprotection. Repeated blood flow restriction and reperfusion in a tissue/organ remote from the heart (remote ischemic conditioning, RIC) reduce myocardial I/R injury and attenuate platelet activation. Whether or not platelets mediate RIC’s cardioprotective signal is currently unclear.

**Methods and Results:**

Venous blood from healthy volunteers (without or with pretreatment of 500/1000 mg aspirin or 180 mg ticagrelor orally, 2–3 h before the study, *n* = 18 each) was collected before and after RIC (3 × 5 min blood pressure cuff inflation at 200 mmHg on the left upper arm/5 min deflation). Washed platelets were isolated. Platelet-poor plasma was used to prepare plasma-dialysates. Platelets (25 × 10^3^/µL) or plasma-dialysates (1:10) prepared before and after RIC from untreated versus aspirin- or ticagrelor-pretreated volunteers, respectively, were infused into isolated buffer-perfused rat hearts. Hearts were subjected to global 30 min/120 min I/R. Infarct size was stained. Infarct size was less with infusion of platelets/plasma-dialysate after RIC (18 ± 7%/23 ± 9% of ventricular mass) than with platelets/plasma-dialysate before RIC (34 ± 7%/33 ± 8%). Aspirin pretreatment abrogated the transfer of RIC’s cardioprotection by platelets (after/before RIC, 34 ± 7%/33 ± 7%) but only attenuated that by plasma-dialysate (after/before RIC, 26 ± 8%/32 ± 5%). Ticagrelor pretreatment induced an in vivo formation of cardioprotective factor(s) per se (platelets/plasma-dialysate before RIC, 26 ± 7%/26 ± 7%) but did not impact on RIC’s cardioprotection by platelets/plasma-dialysate (20 ± 7%/21 ± 5%).

**Conclusion:**

Platelets serve as carriers for RIC’s cardioprotective signal through an aspirin-sensitive and thus cyclooxygenase-dependent mechanism. The P2Y_12_ inhibitor ticagrelor per se induces a humoral cardioprotective signal.

**Graphical abstract:**

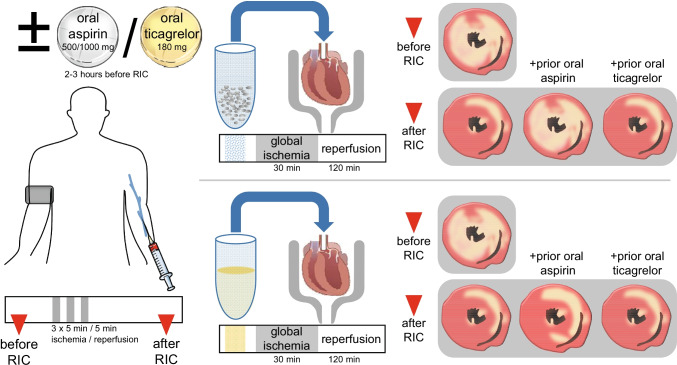

**Supplementary Information:**

The online version contains supplementary material available at 10.1007/s10557-022-07345-9.

## Introduction

Despite substantial improvements of pharmacological and interventional strategies to treat patients with acute myocardial infarction, mortality remains high, with about 15% 1-year mortality in a recent large Scandinavian registry [1]. Thus, even in developed countries with optimal medical treatment and rapid initiation of reperfusion, there is still a need for cardioprotection. Although reperfusion is the only way to rescue myocardium at risk from myocardial infarction, reperfusion per se adds specific irreversible damage to the ischemic injury [2]. So far, cardioprotective strategies beyond that of rapid reperfusion have been developed in preclinical studies but not successfully translated to clinical benefit of patients [3].

Platelet activation exacerbates ischemia/reperfusion (I/R) injury: activated platelets reduce myocardial perfusion through thrombus formation, release vasoconstrictive factors, promote endothelial dysfunction, and trigger inflammation [4]. Pharmacological dual anti-platelet treatment, i.e., the combination of aspirin and a P2Y_12_ inhibitor, is therefore a cornerstone of current therapy of patients undergoing elective or primary percutaneous coronary intervention (PCI) [5], particularly with the aim to prevent thrombosis at the implanted stent surface. However, apart from the deleterious role of activated platelets during myocardial I/R, platelets also exert cardioprotection. Platelets carry and release multiple cardioprotective factors, which can activate intracellular cardioprotective pathways [4, 6, 7]. Indeed, infusion of washed platelets in a subphysiological concentration into an isolated perfused rodent heart improved its functional recovery during reperfusion and reduced infarct size [8, 9]. The potential protective role of platelets during myocardial I/R is supported by a reverse experimental approach: platelet depletion, a most radical anti-platelet intervention, did not reduce infarct size in anesthetized dogs with coronary occlusion/reperfusion [10], possibly because both damage and protection were abrogated. The ability of platelets to store and release cardioprotective factors renders them a potential target for novel cardioprotective strategies [6].

One strategy to induce cardioprotection in acute myocardial infarction consists of brief cycles of I/R in tissues/organs remote from the heart, which then protect the myocardium from sustained I/R injury [3]. Such remote ischemic conditioning (RIC) is operative in all species tested so far and improved patients’ outcome in several but not in all studies [3, 11, 12]. Problems of RIC’s translation into the clinic have been attributed to an incomplete understanding of the complex underlying signal transfer [13] and confounders such as, age, sex, anesthetic regimen, comorbidities, and comedications [14, 15]. RIC is a systemic phenomenon, which requires a stimulus, e.g., I/R cycles in peripheral tissues/organs and a signal transfer to the target organ. The signal transfer of RIC from the periphery to the heart involves humoral and neuronal pathways [16]. Evidence for RIC’s humoral signal transfer was derived from preclinical experiments, where RIC’s cardioprotection was transferred with plasma or plasma-dialysate from conditioned donors to another individual’s isolated heart, which was subjected to ex vivo I/R [17–20]. As a systemic phenomenon, RIC impacts also on circulating blood cells [6]. RIC attenuated platelet activation in patients with coronary artery disease after treadmill exercise [21] or after coronary angiography [22]. In patients undergoing PCI [23] or interventional ablation for atrial fibrillation [24], RIC attenuated platelet-monocyte aggregation [23, 24] and in vitro platelet aggregation in response to adenosine diphosphate (ADP) [24]. RIC increased occlusion time in an in vitro thrombosis test of blood samples taken from ST-segment elevation myocardial infarction patients 48 h after primary PCI [25], reflecting attenuated platelet aggregation. However, while RIC attenuates platelet activation, it is unclear whether or not RIC’s cardioprotective signal transfer involves platelets.

We therefore now studied whether or not platelets serve as transmitters of RIC’s cardioprotective signal. Venous blood was taken from healthy volunteers before and after RIC, respectively. Washed platelets or plasma-dialysates were infused into isolated perfused rat hearts before global I/R. To study the impact of commonly used platelet aggregation inhibitors, the same volunteers were again subjected to RIC after pretreatment with an oral loading dose of either aspirin or ticagrelor, respectively. In the isolated perfused rat hearts, infarct size served as the most robust endpoint of cardioprotection [26, 27].

## Methods

Healthy volunteers were recruited, and experiments were performed between October 2020 and December 2021. The study was approved by the institutional ethical review board (No. 18–8279-BO) of the University of Essen Medical School and conforms to the Declaration of Helsinki. The experimental protocols conform to the guidelines from the Directive 2010/63/EU of the European Parliament on the protection of animals used for scientific purposes. We followed the ARIVE guidelines 2.0. Male Lewis rats (200–380 g, 2.0–3.5 months, Central Animal Laboratory, University of Duisburg-Essen, Essen, Germany) were used in the present study. The experimental protocols in isolated buffer-perfused rat hearts, the plasma-dialysate preparation [19], and the methods for the measurement of hemodynamics and quantification of infarct size were standard [26] and have been described in detail previously [20]. The preparation of venous blood samples and washed platelets was modified from a previous study [28]. Unless otherwise specified, materials were obtained from Sigma-Aldrich (Deisenhofen, Germany).

### Remote Ischemic Conditioning

**Fig. 1:**
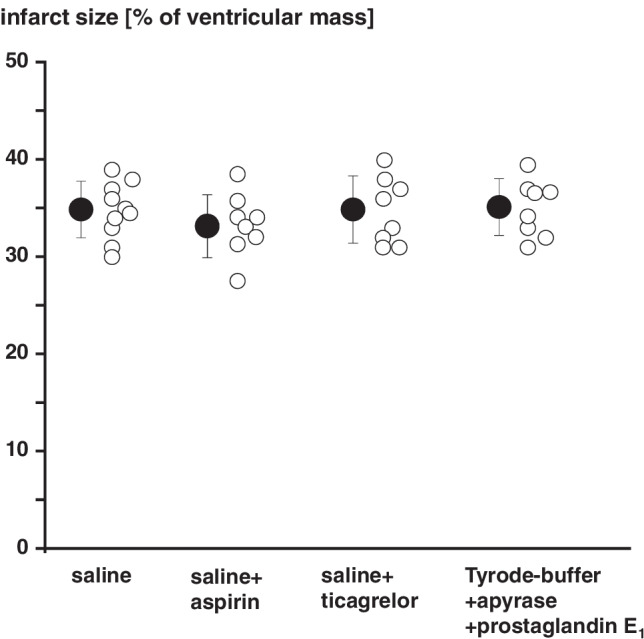
Infusion of saline, aspirin, ticagrelor, or Tyrode buffer supplemented with apyrase and prostaglandin E_1_ does not impact on infarct size. Solutions were infused into isolated perfused rat hearts subjected to 30-min global ischemia and 120-min reperfusion. The concentrations for infusion with aspirin (40 µmol/L, *n* = 8) and ticagrelor (10 µmol/L, *n* = 8) were chosen to reflect the expected maximum plasma concentration 2–3 h after oral ingestion of aspirin and ticagrelor, respectively, in healthy volunteers. The concentration of apyrase and prostaglandin E_1_ (0.1 U/mL and 1 µmol/L, *n* = 8) was equal to that used for the preparation of washed platelet solutions. Infusion of saline (*n* = 10) served as control

**Fig. 2:**
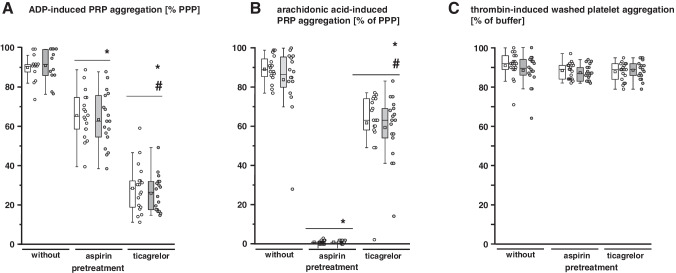
Turbidometric light transmission aggregometry of platelets sampled from volunteers before and after remote ischemic conditioning (RIC). Platelets were sampled before and after RIC from the same volunteers (*n* = 18): without pretreatment (*n* = 18), with aspirin pretreatment (*n* = 18), and with ticagrelor pretreatment (*n* = 18), respectively. White color indicates data before RIC, gray after RIC. PPP, platelet-poor plasma; PRP, platelet-rich plasma. **a **ADP-induced aggregation of platelet-rich plasma. *p < 0.001 vs. without pretreatment; #*p* < 0.001 vs. with aspirin pretreatment; Kruskal–Wallis one-way ANOVA on Ranks and Dunn’s multiple comparison procedures. **b **Arachidonic acid-induced aggregation of platelet-rich plasma. *p < 0.001 vs. without pretreatment; #*p* < 0.001 vs. with aspirin pretreatment; Kruskal–Wallis one-way ANOVA on Ranks and Dunn’s multiple comparison procedures. **c **Thrombin-induced aggregation of washed platelets

The volunteers (9 females/9 males; 31 ± 9 years, 25 ± 3 kg/m^2^ body mass index) had neither a history of disease nor any recent medication, except for oral contraceptives in women, and they were non-smokers. Written informed consent was obtained. Volunteers were not fasted before the experimental protocol. RIC was induced by inflating a blood pressure cuff on the left upper arm to 200 mmHg for 5 min to induce ischemia followed by 5-min reperfusion through rapid deflation of the blood pressure cuff. Three such cycles of upper arm I/R were performed in total. RIC was performed (a) without any prior medication, (b) 3 h after oral ingestion of 500 mg aspirin, and (c) 2 h after oral ingestion of 180 mg ticagrelor. In 6 individuals, the medication with aspirin was repeated with 1000 mg aspirin. Platelets of these individuals induced a different response in terms of impact on infarct size reduction than those of the other individuals (Online Resources Fig. 1). However, platelet aggregations of these 6 individuals were comparable to those of the other individuals after 500 mg aspirin (Online Resources Fig. 2). The experiments with 1000 mg aspirin were performed after analysis of the experiments with 500 mg aspirin. The respective intervals between drug ingestion and the RIC protocol were chosen in order to perform RIC during the expected maximum effect of a single loading dose of aspirin [29] or ticagrelor [30], respectively, on platelet function. An interval of 2 weeks between the RIC protocols was chosen, because cardioprotective factors released in response to RIC persist for up to 8 days in the plasma of healthy volunteers [19]. Before and 60 min after completion of the RIC protocol, the cubital vein at the contralateral arm was punctured, and 80 mL of venous blood was withdrawn into citrated (3.2%) polypropylene tubes (S-Monovette®, Sarstedt, Nümbrecht, Germany). An additional 0.5 mL venous blood sample was used for analyzing the blood cell count (DxH 500, Beckman Coulter, Pasadena, USA) (see Online Resources Table 1).

### Platelet and Plasma-Dialysate Preparation

Venous blood samples were supplemented with apyrase (0.1 U/mL) and prostaglandin E_1_ (1 µmol/L, Tocris Bioscience, Bristol, UK) and centrifuged at 100 × g for 15 min at room temperature. Platelet-rich plasma (PRP) was collected; the remaining blood was centrifuged at 800 × g for 12 min at room temperature to prepare platelet-poor plasma (PPP). PPP used for plasma-dialysate preparation was centrifuged again at 2400 × g for 10 min before being placed into a dialysis tube with a pore size of 12–14 kDa (Spectra Por, Spectrum Europe, Breda, the Netherlands) and dialyzed for 16 h against a tenfold volume of calcium-free Krebs–Henseleit buffer (in mmol/L, NaCl 118.0, KCl 4.7, MgSO_4_ 16.0, KH_2_PO_4_ 1.2, glucose 5.6, sodium pyruvate 2.0) as described previously [19]. The dialysates were titrated to CaCl_2_ 2.0 and 24.0 NaHCO_3_, filtered with a 5-µm pore-sized syringe filter and gassed during pre-warming to 37 °C with 95% O_2_ and 5% CO_2_, pH 7.40 before use. The platelet number in the PRP was determined using light microscopy and counted in a Neubauer chamber. The platelet concentration was adjusted to 250 × 10^3^/µL by adding autologous PPP. An aliquot of the adjusted PRP was taken to confirm attenuation of platelet aggregation after aspirin or ticagrelor pretreatment, respectively, using turbidometric light transmission aggregometry (LTA, two-channel turbidometric aggregometer, Chrono-Log Corporation, Havertown, USA) in response to 5 µmol/L ADP and 250 µg/L arachidonic acid at 37 °C under constant stirring (1200 rpm). Aggregation was measured as area under the curve within 6 min after agonist addition and expressed as percent of the light transmission of PPP which served as control (= 100%). PRP was mixed (1:1) with citrated buffer (in mmol/L, 103.0 NaCl, 5.0 KCl, 5.0 glucose, 0.4 C_6_H_8_O_7_xH_2_O, 0.9 Ca_2_Cl, 3.5 mg/mL fatty acid-free bovine serum albumin (BSA, fraction V, pH adjusted to 6.50 with 2.0 mol/L NaOH, SERVA Electrophoresis GmbH, Heidelberg, Germany)) containing apyrase (0.015 U/mL) and prostaglandin E_1_ (1 µmol/L) and centrifuged at 1000 × g for 12 min at room temperature. The resulting pellet was re-suspended with citrated buffer and again centrifuged at 1000 × g for 12 min at room temperature. The resulting pellet was re-suspended in modified Tyrode buffer (in mmol/L, 130.0 NaCl, 2.6 KCl, 10.0 glucose, 0.4 C_6_H_8_O_7_xH_2_O, 5.0 NaHCO_3_, 10.0 N-2-hydroxyethyl-piperazine-N-2-ethanesulfonic acid, 3.5 mg/mL fatty acid-free BSA (pH adjusted to 7.35 with 2.0 mol/L NaOH)) corresponding to one third of the original PRP volume. The platelet concentration was determined as described above and adjusted to 250 × 10^3^/µL by adding modified Tyrode buffer. The viability of the washed platelets was assessed by the aggregation in response to thrombin (1 U/mL). Again, aggregation was measured as area under the curve within 6 min after agonist addition and expressed as percent of light transmission of Tyrode buffer which served as control (= 100%). The washed platelet solution was placed into a 50-mL polypropylene syringe and warmed to 37 °C under constant gentle stirring with a Teflon stirrer for 15 min before use.

### Isolated Buffer-Perfused Hearts

Rats were euthanized by an intraperitoneal injection of a single lethal dose sodium pentobarbital (800 mg/kg, Narkodorm®, CP-Pharma, Burgdorf, Germany). After disappearance of the withdrawal reflex and immediately with the onset of apnea, beating hearts were rapidly excised within less than 1 min, the aorta cannulated, mounted on a Langendorff apparatus, and perfused with modified Krebs–Henseleit buffer at constant pressure of 65–70 mmHg as described previously [31] (for details, see also Methods in the Online Resources). Coronary flow (CF) and left ventricular developed pressure (LVDP) were continuously recorded, and heart rate was kept at 360 beats per min by right atrial pacing. Hearts were allowed to stabilize for 10–20 min, before baseline values for CF and LVDP were recorded. Specific inclusion and exclusion criteria are detailed in the Supplemental Methods in the Online Resources. Washed platelets were then infused into the aortic cannula using a low adhesive Teflon tubing at a flow rate which substituted 10% of the measured CF flow rate for 8 min, followed by a 2 min washout period. Plasma-dialysates were also infused for 8 min, followed by a 2 min washout period. Hearts were then subjected to 30 min/120 min global I/R. After completion of the experimental protocol, hearts were frozen at − 20 °C in Cryomatrix™ (Thermo Fisher Scientific, Schwerte, Germany) and cut into transverse 2-mm-thick slices. Infarct size was demarcated by triphenyl tetrazolium chloride staining, calculated as percent of the sum of left and right ventricular mass and expressed in percent of ventricular mass. The recovery of LVDP during reperfusion is inversely related to infarct size; however, during the short observation period of our reperfusion protocol, this relationship is confounded by stunning [26, 32]. Therefore, we used only infarct size as endpoint of cardioprotection.

In preliminary experiments, infusion of saline, saline supplemented with 40 µmol/L aspirin, saline supplemented 10 µmol/L ticagrelor, or infusion of Tyrode buffer supplemented with 0.1 U/mL apyrase and 1 µmol/L prostaglandin E_1_ served as controls. The concentrations for aspirin and ticagrelor were chosen to reflect the expected maximum plasma concentration 2–3 h after oral ingestion of aspirin [29] or ticagrelor [30], respectively, in fasted volunteers. Apyrase and prostaglandin E_1_ were added to Tyrode buffer in equal concentrations to those used for platelet preparation. There were neither differences in the recovery of CF and LVDP (see Online Resources Table 2) nor in infarct size (Fig. 1) with infusion of saline, saline supplemented with aspirin, saline supplemented with ticagrelor, or Tyrode buffer supplemented with apyrase and prostaglandin E_1_, respectively. Block randomization was used to allocate isolated perfused rat heart preparations to infusion of saline solutions, washed platelets, or plasma-dialysates.

### Statistics

Investigators performing the experiments in isolated buffer-perfused heart and analyzing infarct size and time courses of CF and LVDP in isolated buffer-perfused heart and platelet aggregation in response to agonists were blinded with respect to protocol and pretreatment in the volunteers (before/after RIC ± aspirin or ± ticagrelor, respectively). Investigators analyzing data sets were blinded with respect to all protocols and pretreatment. Investigators who performed RIC in volunteers and volunteers who received RIC could not be blinded, since RIC requires inflation/deflation of the blood pressure cuff. The Shapiro–Wilk test was used to test for normal distribution of all data. The assumption of normal distribution was confirmed for all analyzed data sets, except for the platelet aggregation data obtained with LTA. Data are presented as means ± standard deviations or as median [interquartile range]. One-way ANOVA was used to analyze CF and LVDP at baseline between all hearts, infarct size in isolated buffer-perfused rat hearts infused with saline, saline supplemented with aspirin or ticagrelor, or Tyrode buffer supplemented with apyrase and prostaglandin E_1_. Time courses of CF and LVDP in isolated buffer-perfused rat hearts were analyzed by two-way (time, protocol: before versus after RIC) ANOVA for repeated measures. Two-way ANOVA for repeated measures was also used to analyze infarct size in isolated buffer-perfused rat hearts with infusion of washed platelets or plasma-dialysates (without pretreatment or with aspirin/ticagrelor, protocol: before versus after RIC), respectively. Individual mean values were compared by Fisher’s least significant difference post hoc tests when ANOVA indicated a significant difference. One-way Kruskal–Wallis ANOVA on ranks with Dunn’s multiple comparison procedure was used to analyze the degree of aggregation in LTA. Differences were considered significant at the level of *p* < 0.05 (SigmaStat 3.5, Erkrath, Germany), and exact *p* values are given for *p* values when ≥ 0.001 for infarct size and platelet aggregation.

## Results

### Blood Cell Count and Platelet Function

Blood cell counts were comparable between blood samples taken before or after RIC, irrespectively of the pretreatment with aspirin and ticagrelor, respectively (Online Resources Table 1). With addition of ADP or arachidonic acid, PRP aggregation was not different in preparations from blood taken before and after RIC (Fig. 2). Both aspirin pretreatment and ticagrelor pretreatment reduced the ADP-induced PRP aggregation (Fig. 2a). Aspirin pretreatment abrogated and ticagrelor pretreatment attenuated the arachidonic acid-induced PRP aggregation (Fig. 2b). In washed platelets used for infusion into isolated hearts, the aggregation induced by 1 U/mL thrombin was not different before and after RIC nor did aspirin pretreatment or ticagrelor pretreatment impact on it (Fig. 2c), reflecting full aggregatory functionality of washed platelets.

### Coronary Flow, Left Ventricular Function, and Infarct Size in Isolated Perfused Rat Hearts

In isolated perfused rat hearts, baseline CF and LVDP were not different between hearts at baseline. The infusion of washed platelets — independently of whether they were taken before or after RIC — induced a transient reduction of CF and LVDP with recovery during washout before ischemia. Also, the CF recovery after infusion of washed platelets was comparable between all hearts. However, the recovery of LVDP during reperfusion was better after infusion of washed platelets from volunteers with prior aspirin or ticagrelor pretreatment than with infusion of platelets from volunteers without pretreatment (Online Resources Table 2). The infusion of plasma-dialysates, prepared from samples taken before or after RIC, induced a slight increase in CF and a transient reduction of LVDP. LVDP recovered fully during washout — irrespectively of whether volunteers were pretreated with aspirin or ticagrelor or not. The CF and LVDP recoveries during reperfusion were comparable between hearts infused with plasma-dialysate before and after RIC without or with aspirin pretreatment. The recovery of LVDP was improved with infusion of plasma-dialysates after ticagrelor pretreatment (Online Resources Table 2).

**Fig. 3:**
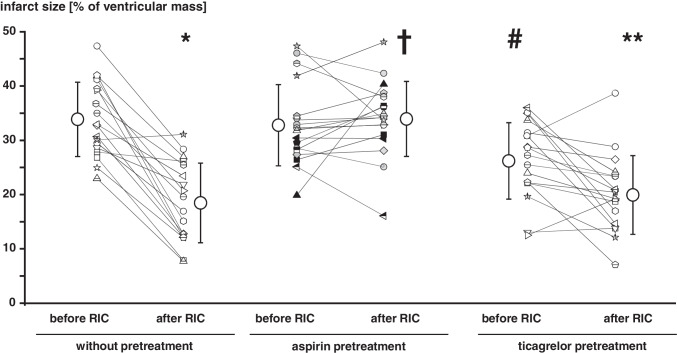
Platelets serve as transmitters of remote ischemic conditioning’s (RIC)s cardioprotective signal through an aspirin-sensitive mechanism. Platelets were sampled before and after RIC from the same volunteers (*n* = 18): without pretreatment (*n* = 18), with aspirin pretreatment (*n* = 18), and with ticagrelor pretreatment (*n* = 18), respectively. Platelets were infused into isolated perfused rat hearts, which were then subjected to 30-min global ischemia/120-min reperfusion. In the aspirin pretreatment group, gray symbols indicate volunteers who were pretreated with 500 mg aspirin (*n* = 12) and black symbols those with 1000 mg aspirin (*n* = 6). *p < 0.001 vs. before RIC without pretreatment; **p < 0.002 vs. before RIC with ticagrelor pretreatment; #*p* = 0.002 vs. before RIC without pretreatment and *p* = 0.011 vs. before RIC with aspirin pretreatment; †*p* < 0.001 vs. after RIC without pretreatment and vs. after RIC with ticagrelor pretreatment; two-way repeated measures ANOVA with Fisher’s least significant differences post hoc tests

**Fig. 4:**
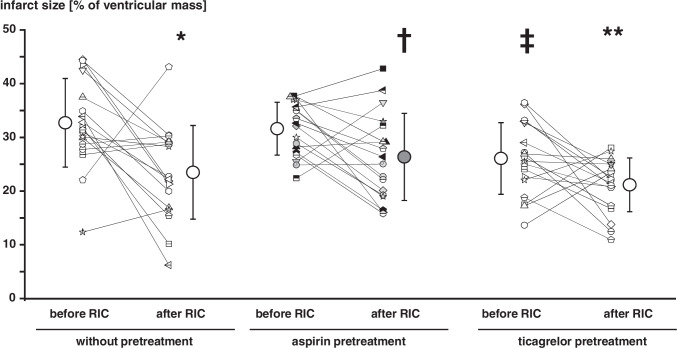
Pretreatment with aspirin or ticagrelor does not impact on the cardioprotective transfer of remote ischemic conditioning (RIC) via plasma-dialysate. Plasma-dialysates were prepared before and after RIC from the same volunteers (*n* = 18): without pretreatment (*n* = 18), with aspirin pretreatment (*n* = 18), and with ticagrelor pretreatment (*n* = 18), respectively. Plasma-dialysates before and after RIC were infused into isolated perfused rat hearts, respectively, which were then subjected to 30-min global ischemia/120 min reperfusion. In the aspirin pretreatment group, gray symbols indicate volunteers who were pretreated with 500 mg aspirin (*n* = 12) and black symbols (*n* = 6) those with 1000 mg aspirin.*p < 0.001 vs. before RIC without pretreatment; †*p* = 0.030 vs. before RIC with aspirin pretreatment; **p = 0.044 vs. before RIC with ticagrelor pretreatment and *p* = 0.041 vs. after RIC with aspirin pretreatment; ‡*p* = 0.010 vs. before RIC without pretreatment and *p* = 0.029 vs. before RIC with aspirin pretreatment; two-way repeated measures ANOVA with Fisher least significant differences post hoc tests

Infusion of washed platelets before RIC had no impact on infarct size per se (34 ± 7%) when compared to saline (35 ± 3%), and, again, aspirin and ticagrelor when added to the isolated perfused heart were not cardioprotective per se (Fig. 1). Infusion of washed platelets after RIC reduced infarct size to 18 ± 7% (Fig. 3). Infusion of washed platelets from aspirin-pretreated volunteers did not impact on infarct size before RIC (33 ± 7%) (Fig. 3) but abrogated the infarct size reduction after RIC (34 ± 7%) (Fig. 3). However, ticagrelor when given systemically to healthy volunteers induced the formation of cardioprotective factor(s) which were transferable to the isolated perfused heart. Infusion of washed platelets from ticagrelor-pretreated volunteers before RIC reduced infarct size per se (26 ± 7%) (Fig. 3), whereas infusion of washed platelets after RIC reduced infarct size to the same level in presence (20 ± 7%) as in the absence of ticagrelor (18 ± 7%) (Fig. 3). Infusion of plasma-dialysate before RIC had no impact on infarct size per se (33 ± 8%) when compared to saline (35 ± 3%). Infusion of plasma-dialysate after RIC reduced infarct size to 23 ± 9% (Fig. 4). Plasma-dialysate from aspirin-pretreated volunteers did not impact on infarct size per se (32 ± 5%) (Fig. 4). However, different to infusion of washed platelets after RIC, aspirin pretreatment of volunteers did not abrogate the infarct size reduction by plasma-dialysate after RIC (26 ± 8%) (Fig. 4). As with infusion of washed platelets, the infusion of plasma-dialysate from ticagrelor-pretreated volunteers before RIC reduced infarct size per se (26 ± 7%) (Fig. 4), and ticagrelor pretreatment did not impact on the infarct size reduction achieved by plasma-dialysate after RIC (21 ± 5%) (Fig. 4).

## Discussion

**Fig. 5:**
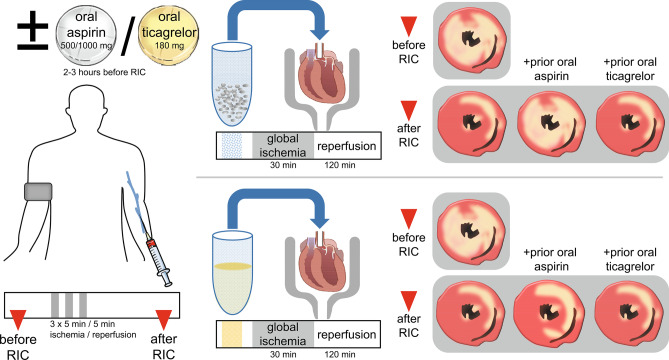
In response to remote ischemic conditioning (RIC), platelets transfer a cardioprotective signal through an aspirin and thus Cox-sensitive mechanism. Aspirin pretreatment of healthy volunteers abrogated RIC’s transfer of cardioprotection through washed platelets but not that through plasma-dialysate, which supports a platelet-specific component of RIC’s humoral transfer of cardioprotection. Ticagrelor pretreatment obviously did not impact on protection by RIC, since the level of infarct size reduction achieved with platelets or plasma-dialysate from blood after RIC was not different in the absence or presence of ticagrelor. Washed platelets or plasma-dialysates from volunteers before and after RIC (without or with aspirin/ticagrelor pretreatment) were infused into an isolated perfused rat heart with 30-min global ischemia and 120-min reperfusion with infarct size as endpoint

Our study supports the notion that platelets carry and release cardioprotective factor(s) which reduce rather than promote myocardial I/R injury [4, 6, 7]. Platelets contributed to the humoral signal transfer of RIC’s cardioprotection. RIC’s cardioprotection was transferred via washed platelets from healthy volunteers with RIC to isolated perfused rat hearts (Fig. 5). Different from previous studies, where infusion of platelets in a subphysiological concentration induced cardioprotection [8, 9], infusion of washed platelets in our study did not impact on infarct size per se. Subtle differences in the timing of administration and concentration of platelets may account for such difference. There is one prior study, indicating a potential role of platelets for RIC’s signal transfer and protection of the liver. In a mouse model with hepatic I/R, thrombocytopenia through infusion of anti-cluster of differentiation 41 immunoglobulin abrogated RIC’s protection [33], supporting a potential role for platelets in RIC’s signal transfer, but that study was possibly also confounded by other effects of thrombocytopenia [34]. As in our previous studies [19, 20, 35], RIC’s cardioprotection was also transferable with plasma-dialysate (Fig. 5). The observation that aspirin pretreatment abrogated RIC’s transfer of cardioprotection through platelets but not that through plasma-dialysate supports a platelet-specific component of RIC’s humoral transfer of cardioprotection (Fig. 5). However, we looked at the inhibition of cardioprotection by aspirin only in a qualitative/binary (yes/no) fashion. Although 500 mg aspirin effectively abrogated arachidonic acid-induced platelet aggregation in all volunteers, in six volunteers, only 1000 mg aspirin sufficiently abrogated RIC’s transfer of cardioprotection with platelets. Thus, it appears that there are quantitative differences between aspirin’s inhibition of platelet aggregation and inhibition of cardioprotection by platelets. Abrogation of RIC’s platelet-mediated transfer of cardioprotection by aspirin pretreatment in our study suggests a cyclooxygenase (Cox)-dependent signaling. Doses > 300 mg aspirin (we used 500/1000 mg) inhibit Cox-1 [36], which is predominant in platelets [37], but also Cox-2, which is induced in response to inflammatory stimuli in various tissues but also constitutively expressed in neuronal tissue. In platelets, prostaglandin G_2_ formation is Cox-1 dependent, and prostaglandin G_2_ serves as a common precursor for the potent platelet agonist thromboxane A_2_ and for additional prostaglandins and prostacyclins. There is evidence for several prostaglandin species to mediate cardioprotection. Endogenous prostaglandins I_2_ [38, 39], D_2_ [40], and E_2_ [41–43] contributed to cardioprotection through the activation of prostaglandin receptors 3 and 4 in different species with I/R. To dissect which specific prostaglandin/prostaglandin receptor is involved in our setup would require use of selective pharmacological antagonists. The possible nonspecific nature of such agents [44] and their off-target effects [45], however, made such approach not feasible within the framework of our current study.

The constitutively expressed Cox-2 is relevant for spinal nociceptive signaling [46]. Of note, RIC’s signal transfer from the periphery to the target organ heart involves activation of peripheral sensory nerves, which project into autonomic centers of the central nervous system and consequently the vagal nerves [16, 20]. Activation of the vagal nervous system results in a release of humoral cardioprotective factors from the spleen [20] and other abdominal organs [16]. Thus, not only the platelet-dependent Cox signaling but also that in the periphery may have been affected by aspirin. However, again, in our study, aspirin pretreatment only abrogated the transfer of cardioprotection by platelets, while plasma-dialysate still mediated cardioprotection, and, consequently, a platelet-specific Cox-sensitive component of RIC’s cardioprotection is more likely. Also, aspirin in prior studies had abolished the cardioprotection by local ischemic preconditioning [47] and postconditioning [48] when given in vivo in rodent models with regional I/R. The attenuation of ischemic conditioning’s cardioprotection in these studies was attributed to the inhibition of myocardial Cox-2 activity [49]. In our study, any direct impact of aspirin on the rat myocardium and thus on infarct size seems unlikely, since platelets were repeatedly washed, resulting in an estimated dilution of hypothetical contaminating plasma by 10^8^ and since infusion of aspirin in saline control experiments had no impact on infarct size. Nevertheless, in vivo aspirin may not only impact on the platelet-mediated transfer of cardioprotection but also on the myocardial responsiveness to cardioprotective signaling. Taken together, it is virtually impossible to attribute the aspirin-mediated abrogation of cardioprotection by RIC exclusively to the inhibition of platelet-dependent Cox-1 or to inhibition of Cox-2 signaling in the periphery or the myocardium in our and the above studies.

Ticagrelor exerts cardioprotective properties per se [50, 51]. Our data support the cardioprotective potential of ticagrelor: the cardioprotective factor(s) recruited by ticagrelor pretreatment in vivo reduced infarct size in the isolated perfused heart per se. Platelets, and interestingly, also plasma-dialysate, prepared from the same volunteers after ticagrelor pretreatment, reduced infarct size in the isolated perfused rat heart. Whether or not platelets were the origin of such plasmatic cardioprotective factor(s), however, remains unclear. Nevertheless, a systemic administration of ticagrelor appears to be mandatory to exert its cardioprotective effect. Not only in the present study, but also in prior studies, ticagrelor did not impact on infarct size in isolated perfused rat hearts [51, 52]. In vivo, ticagrelor increases the systemic circulating adenosine levels [53, 54], which can activate cardioprotective pathways [3]. Such ticagrelor-mediated increase in systemic adenosine was attributed to the inhibition of the equilibrative nucleoside transporter-1, which results in a decreased adenosine uptake by erythrocytes [55] and thus increased local adenosine levels in ischemic tissues [50]. Such prolongation of adenosine’s half-life may subsequently enhance adenosine receptor-mediated cardioprotection [50]. Therefore, platelet-mediated — but also plasma-mediated — cardioprotection after in vivo ticagrelor pretreatment in our study could be related to a yet unknown effect of ticagrelor on circulating platelets, hypothetically impacting on adenosine metabolism within platelets and erythrocytes [56, 57]. In line with this notion, platelet-derived adenosine reduced infarct size in isolated perfused rat hearts [56]. However, ticagrelor pretreatment obviously did not impact on protection by RIC, since the level of infarct size reduction achieved with platelets or plasma-dialysate from blood after RIC was not different in the absence or presence of ticagrelor (Fig. 5). Interestingly, in an in vivo rat model of myocardial infarction, ticagrelor pretreatment was also not additive to the cardioprotection afforded by ischemic postconditioning — although cardioprotective per se [58]. The lack of an additive action of ticagrelor may reflect the use of the same cardioprotective signal transduction cascade by ticagrelor and RIC. Apart from and in addition to the above platelet-mediated cardioprotective pathways, platelets may also contribute to the humoral signal transfer of cardioprotection via released extracellular vesicles, which contain cardioprotective microRNAs [59, 60]. In response to RIC specifically, microRNA144-3p and microRNA451a were increased in the circulating extracellular vesicles of healthy humans [60].

Anti-platelet drugs have repeatedly been discussed as potential confounders of RIC’s cardioprotection in patients with acute myocardial infarction [15]. However, there is currently no study analyzing whether or not anti-platelet drugs interfere with RIC’s cardioprotection. In studies on RIC’s impact on the outcome of patients undergoing PCI for the treatment of acute ST-segment elevation myocardial infarction, virtually, all patients received a loading dose of aspirin in temporal relation to PCI [61]. Among those studies, however, there are some reporting cardioprotection by RIC, whereas others reported neutral results [61]. The largest multicenter study on RIC’s cardioprotection in patients with ST-segment elevation myocardial infarction, the CONDI-2/ERIC-PPCI trial, failed to demonstrate a reduction of infarct size or an improved outcome [62]. Recently, Ye et al. [63] hypothetically proposed that aspirin may attenuate cardioprotection by RIC. Our data now clearly demonstrate that indeed aspirin abrogates the platelet-mediated part of RIC’s cardioprotective signal. Thus, in clinical studies, differences between the placebo and RIC groups may have been obscured by aspirin. As outlined above, ticagrelor has pleiotropic cardioprotective effects beyond that of platelet inhibition and also reduces infarct size in patients [64]. Approximately 70% of all patients in both, the placebo and the RIC arm of the CONDI-2/ERIC-PPCI trial, received ticagrelor in temporal relation to PCI, and indeed, in a post hoc subgroup analysis of the CONDI-2 trial, ticagrelor-pretreated patients had an improved clinical outcome compared to those pretreated with clopidogrel or prasugrel [65]. Of note, in a previous study with a similar setting conducted in the pre-ticagrelor era, where 95–97% of patients still had received clopidogrel in temporal relation to PCI, RIC did reduce infarct size [66, 67]. The use of dual platelet inhibition with aspirin and ticagrelor in clinical trials on novel cardioprotective interventions may then impair cardioprotection in the treatment group by aspirin and recruit some cardioprotection per se in the placebo group by ticagrelor, such that the difference between the treatment and placebo group induced by the cardioprotective intervention under study is minimized — exactly that was probably true in the ERIC-PPCI/ CONDI 2 trial.

### Limitations

We have recruited healthy and young volunteers of both sexes; however, age, comorbidities, and comedications clearly impact on platelet function and may also impact on the platelet-mediated signal transfer of RIC. Whether or not long-term low-dose aspirin — as often seen in patients with manifest coronary artery disease — also interferes with RIC’s cardioprotection remains to be seen. We did not focus on the potential impact of RIC on platelet aggregation, and more sensitive methods than LTA are available to study the potentially delicate effect of RIC on platelet aggregation and function. Also, any labile anti-platelet factor, such as nitric oxide, which is released by RIC [16, 68] likely has disappeared in our in vitro preparations but might have had consequences for platelet activity in vivo. In our setup, we diluted the washed platelets by a factor of 10 compared to the platelet number in the circulating blood. Thus, the magnitude of the cardioprotective transfer via platelets may have been underestimated. The observation that aspirin pretreatment abolished exclusively the platelet transfer of RIC's cardioprotection is restricted to our experimental setup with the focus on RIC’s humoral transfer. Future studies are needed to more exactly determine dose–response and temporal relationships for aspirin’s impact on RIC’s cardioprotection. We here chose an aspirin dose, which only roughly resembles that of a patient who is already taking oral low-dose aspirin and receives an additional loading dose in a setting of PCI.

## Conclusion and Future Perspective

Platelets are not only a target of RIC, but also transmit RIC’s cardioprotective signal through a Cox-dependent mechanism. Despite the unequivocal detrimental impact of platelet activation during myocardial I/R injury, patients could benefit from a more targeted approach of anti-platelet drugs. Aspirin interferes with the transfer of cardioprotective factors through platelets. In contrast, ticagrelor induces the formation of cardioprotective factors which are carried by platelets and plasma. Therefore, in clinical trials, the use of dual platelet inhibition with aspirin and ticagrelor may obscure the effect of the cardioprotective intervention under study. Finally, to reduce the discrepancy between preclinical and clinical studies (i.e., the influence of comedications and/or comorbidities), the present study further highlights the need to develop animal models which more closely resemble the pathophysiological and pharmacological background of patients[69].

### Supplementary Information

Below is the link to the electronic supplementary material.

Supplementary file1 (DOCX 71 kb)

## Data Availability

All data of the present study are available in the article and its Online Resources. Original data will be shared on reasonable request to the corresponding author.
