# Supplementary material for: Platelet-Mediated Transfer of Cardioprotection by Remote Ischemic Conditioning and Its Abrogation by Aspirin But Not by Ticagrelor
Source: Cardiovasc Drugs Ther. 2022 May 21;37(5):865–76. doi: 10.1007/s10557-022-07345-9 (PMC10517043; doi:10.1007/s10557-022-07345-9)
Supplement: Supplementary file 1 — Supplementary file1 (DOCX 71 kb) [file 10557_2022_7345_MOESM1_ESM.docx]

**SUPPLEMENTARY MATERIAL**

**Platelet-mediated transfer of cardioprotection by remote ischemic conditioning and its abrogation by aspirin, but not by ticagrelor**

Helmut Raphael Lieder^1^, Maria Tsoumani^2^, Ioanna Andreadou^2^, Karsten Schrör^3^, Gerd Heusch^1^, Petra Kleinbongard^1^

^1^Institute for Pathophysiology, West German Heart and Vascular Center, University of Essen Medical School, Essen, Germany

^2^Laboratory of Pharmacology, National and Kapodistrian University of Athens, Athens, Greece

^3^Department of Pharmacology and Clinical Pharmacology, Heinrich-Heine-University Düsseldorf, Düsseldorf, Germany

**Online Resources Figure 1: Differences in Infarct size reduction by infusion of washed platelets from healthy volunteers with remote ischemic conditioning and 500 mg aspirin-pretreatment.** Platelets were sampled before and after RIC from n=18 volunteers: Platelets sampled before and after RIC were infused into isolated perfused rat hearts, which were then subjected to 30 min global ischemia/ 120 min reperfusion. Black symbols indicate samples of those volunteers who received 1000 mg aspirin in the experiments reported in the main manuscript (Figure 3); *p=0.015 vs. before RIC; two-way repeated measures ANOVA with Fisher‘s least significant differences post-hoc tests.

**Online Resources Figure 2: Attenuated aggregation of platelets sampled from volunteers before and after remote ischemic conditioning (RIC) after pretreatment with 500 mg aspirin; A: ADP-induced aggregation of platelet-rich plasma;** **B: arachidonic acid-induced aggregation of platelet-rich plasma; C: thrombin-induced aggregation of washed platelets.** Platelets were sampled before and after RIC from n=18 volunteers**.** Platelet aggregation was measured by turbidometric light transmission aggregometry. Black symbols indicate those volunteers who received 1000 mg aspirin in the experiments reported in the main manuscript (Figure 2) PPP: platelet-poor plasma; PRP: platelet-rich plasma. A: ADP-induced aggregation of platelet-rich plasma. B: Arachidonic acid-induced aggregation of platelet-rich plasma. C: Thrombin-induced aggregation of washed platelets.

**Online Resources Table 1:** Blood cell count of 18 healthy volunteers before and after remote ischemic conditioning without pretreatment, with aspirin-pretreatment or with ticagrelor-pretreatment, respectively

| Groups | ERY (x10^6^/μL) | HGB (g/dL) | HCT  (%) | LEU (x10^3^/μL) | NE (x10^3^/μL) | LY (x10^3^/μL) | MO (x10^3^/μL) | PL  (x10^3^/μL) |
| --- | --- | --- | --- | --- | --- | --- | --- | --- |
|  |  |  |  |  |  |  |  |  |
| before RIC | 4.8±0.4 | 14.4±1.1 | 43.5±3.2 | 5.3±1.7 | 2.9±1.2 | 1.8±0.5 | 0.4±0.2 | 249.9±38.8 |
| after RIC | 4.7±0.4 | 14.2±1.0 | 42.5±3.0 | 5.8±1.9 | 3.5±1.6 | 1.7±0.5 | 0.4±0.1 | 250.7±45.4 |
| before RIC with aspirin-pretreatment | 4.7±0.3 | 13.9±1.0 | 42.2±2.9 | 5.2±1.2 | 2.9±1.0 | 1.7±0.5 | 0.4±0.1 | 261.6±43.8 |
| after RIC with aspirin-  pretreatment | 4.6±0.3 | 13.7±1.0 | 41.2±2.8 | 5.5±1.4 | 3.5±1.2 | 1.5±0.4 | 0.5±0.2 | 257.9±43.9 |
| before RIC with ticagrelor-pretreatment | 4.7±0.3 | 13.9±1.0 | 42.2±2.6 | 5.5±1.6 | 3.1±1.4 | 1.8±0.5 | 0.4±0.1 | 259.0±50.0 |
| after RIC with ticagrelor-pretreatment | 4.7±0.3 | 13.8±1.1 | 41.6±2.8 | 5.5±1.5 | 3.3±1.2 | 1.8±0.5 | 0.4±0.1 | 258.4±49.7 |

Values are means±standard deviations. ERY: erythrocytes; HCT: hematocrit; HGB: hemoglobin; LEU: leukocytes; LY: lymphocytes; MO: monocytes; NE: neutrophils; PL: platelets; RIC: remote ischemic conditioning

**Online Resources Table 2: CF and LVDP of isolated buffer-perfused rat hearts**

|  |  | CF | | | LVDP | | |
| --- | --- | --- | --- | --- | --- | --- | --- |
|  | **time point** | **[mL/min]** | | | **[mmHg]** | | |
| saline (n=10) | baseline | 15.6 | ± | 1.5 | 92 | ± | 10 |
|  | infusion | 15.2 | ± | 1.1 | 95 | ± | 9* |
|  | washout | 15.2 | ± | 1.2 | 94 | ± | 7 |
|  | isch5 | 0.0 | ± | 0.0* | 1 | ± | 0* |
|  | isch25 | 0.0 | ± | 0.0* | 1 | ± | 1* |
|  | rep10 | 8.3 | ± | 2.6* | 17 | ± | 24* |
|  | rep30 | 8.8 | ± | 2.5* | 22 | ± | 29* |
|  | rep60 | 8.7 | ± | 2.5* | 26 | ± | 25* |
| saline+aspirin (n=8) | baseline | 13.3 | ± | 1.4 | 118 | ± | 18 |
|  | infusion | 13.5 | ± | 1.7 | 117 | ± | 20 |
|  | washout | 13.6 | ± | 1.8 | 115 | ± | 20 |
|  | isch5 | 0.0 | ± | 0.0* | 1 | ± | 0* |
|  | isch25 | 0.0 | ± | 0.0* | 1 | ± | 1* |
|  | rep10 | 8.2 | ± | 1.6* | 13 | ± | 9* |
|  | rep30 | 9.4 | ± | 2.0* | 30 | ± | 14* |
|  | rep60 | 8.7 | ± | 1.4* | 37 | ± | 9* |
| saline+ticagrelor (n=8) | baseline | 14.9 | ± | 1.8 | 100 | ± | 13 |
|  | infusion | 15.3 | ± | 1.8 | 99 | ± | 12* |
|  | washout | 15.5 | ± | 2.1 | 98 | ± | 13 |
|  | isch5 | 0.0 | ± | 0.0* | 1 | ± | 0* |
|  | isch25 | 0.0 | ± | 0.0* | 4 | ± | 4* |
|  | rep10 | 7.6 | ± | 2.6* | 8 | ± | 11* |
|  | rep30 | 8.0 | ± | 3.1* | 20 | ± | 15* |
|  | rep60 | 8.7 | ± | 3.0* | 25 | ± | 10* |
| Tyrode-buffer+apyrase+ prostaglandin E_1_ (n=8) | baseline | 16.3 | ± | 1.7 | 99 | ± | 19 |
|  | infusion | 18.2 | ± | 1.4*^α^ | 84 | ± | 16* |
|  | washout | 18.6 | ± | 1.8*^α^ | 90 | ± | 22 |
|  | isch5 | 0.0 | ± | 0.0* | 1 | ± | 1* |
|  | isch25 | 0.0 | ± | 0.0* | 3 | ± | 2* |
|  | rep10 | 8.5 | ± | 2.8* | 10 | ± | 13* |
|  | rep30 | 9.2 | ± | 2.4* | 15 | ± | 20* |
|  | rep60 | 11.5 | ± | 4.4* | 21 | ± | 13* |
|  |  |  |  |  |  |  |  |
| washed platelets before RIC without pretreatment (n=18) | baseline | 15.2 | ± | 1.5 | 98 | ± | 11 |
|  | infusion | 12.0 | ± | 3.2* | 74 | ± | 19* |
|  | washout | 15.5 | ± | 2.1 | 89 | ± | 11 |
|  | isch5 | 0.0 | ± | 0.0* | 1 | ± | 0* |
|  | isch25 | 0.0 | ± | 0.0* | 2 | ± | 3* |
|  | rep10 | 8.5 | ± | 2.0* | 11 | ± | 10* |
|  | rep30 | 9.6 | ± | 1.8* | 19 | ± | 14* |
|  | rep60 | 9.3 | ± | 1.5* | 27 | ± | 15* |
| washed platelets after RIC without pretreatment (n=18) | baseline | 15.2 | ± | 1.2 | 101 | ± | 14 |
|  | infusion | 13.1 | ± | 2.4* | 81 | ± | 18* |
|  | washout | 15.4 | ± | 2.7 | 93 | ± | 22 |
|  | isch5 | 0.0 | ± | 0.0* | 1 | ± | 0* |
|  | isch25 | 0.0 | ± | 0.0* | 2 | ± | 3* |
|  | rep10 | 9.0 | ± | 2.5* | 11 | ± | 14* |
|  | rep30 | 10.0 | ± | 2.5* | 21 | ± | 16* |
|  | rep60 | 9.8 | ± | 2.3* | 29 | ± | 19* |
| washed platelets before RIC with aspirin-pretreatment (n=18) | baseline | 15.4 | ± | 1.5 | 108 | ± | 12 |
|  | infusion | 12.4 | ± | 1.8* | 83 | ± | 19*# |
|  | washout | 16.0 | ± | 2.1 | 102 | ± | 16 |
|  | isch5 | 0.0 | ± | 0.0* | 1 | ± | 0* |
|  | isch25 | 0.0 | ± | 0.0* | 1 | ± | 1* |
|  | rep10 | 9.7 | ± | 3.0* | 25 | ± | 19*#† |
|  | rep30 | 10.6 | ± | 2.9* | 30 | ± | 21*# |
|  | rep60 | 10.7 | ± | 2.7* | 34 | ± | 21*# |
| washed platelets after RIC with aspirin-pretreatment (n=18) | baseline | 14.7 | ± | 1.6 | 104 | ± | 14 |
|  | infusion | 12.5 | ± | 2.7* | 80 | ± | 25* |
|  | washout | 15.4 | ± | 1.8 | 95 | ± | 20 |
|  | isch5 | 0.0 | ± | 0.0* | 1 | ± | 0* |
|  | isch25 | 0.0 | ± | 0.0* | 1 | ± | 1* |
|  | rep10 | 8.8 | ± | 2.8* | 19 | ± | 21*#† |
|  | rep30 | 9.1 | ± | 2.5* | 29 | ± | 25* |
|  | rep60 | 9.5 | ± | 2.3* | 38 | ± | 20*# |
| washed platelets before RIC with ticagrelor-pretreatment (n=18) | baseline | 14.6 | ± | 1.6 | 102 | ± | 12 |
|  | infusion | 11.3 | ± | 2.0* | 76 | ± | 15*# |
|  | washout | 15.9 | ± | 1.7 | 90 | ± | 10 |
|  | isch5 | 0.1 | ± | 0.3* | 1 | ± | 1* |
|  | isch25 | 0.1 | ± | 0.3* | 3 | ± | 5* |
|  | rep10 | 9.5 | ± | 2.4* | 22 | ± | 18*#† |
|  | rep30 | 9.9 | ± | 2.3* | 37 | ± | 19*#† |
|  | rep60 | 9.9 | ± | 1.9* | 40 | ± | 15*#† |
| washed platelets after RIC with ticagrelor-pretreatment (n=18) | baseline | 14.9 | ± | 1.3 | 104 | ± | 15 |
|  | infusion | 12.0 | ± | 2.3* | 79 | ± | 14* |
|  | washout | 15.5 | ± | 1.9 | 93 | ± | 15 |
|  | isch5 | 0.0 | ± | 0.0* | 1 | ± | 1* |
|  | isch25 | 0.0 | ± | 0.0* | 2 | ± | 2* |
|  | rep10 | 9.2 | ± | 2.3* | 21 | ± | 14*#† |
|  | rep30 | 9.8 | ± | 2.2* | 38 | ± | 15*#† |
|  | rep60 | 10.3 | ± | 2.5* | 39 | ± | 14*#† |
|  |  |  |  |  |  |  |  |
| plasma dialysate before RIC without pretreatment (n=18) | baseline | 15.5 | ± | 1.9 | 108 | ± | 14 |
|  | infusion | 16.4 | ± | 1.6* | 87 | ± | 15* |
|  | washout | 16.9 | ± | 1.7* | 97 | ± | 14 |
|  | isch5 | 0.0 | ± | 0.0* | 2 | ± | 1* |
|  | isch25 | 0.0 | ± | 0.0* | 4 | ± | 1* |
|  | rep10 | 10.4 | ± | 2.1* | 18 | ± | 17* |
|  | rep30 | 11.3 | ± | 2.1* | 27 | ± | 18* |
|  | rep60 | 10.7 | ± | 1.8* | 34 | ± | 17* |
| plasma dialysate after RIC without pretreatment (n=18) | baseline | 15.3 | ± | 1.5 | 99 | ± | 12 |
|  | infusion | 16.8 | ± | 2.2* | 79 | ± | 24*# |
|  | washout | 16.9 | ± | 2.4* | 90 | ± | 12 |
|  | isch5 | 0.0 | ± | 0.0* | 1 | ± | 1* |
|  | isch25 | 0.0 | ± | 0.0* | 1 | ± | 1* |
|  | rep10 | 9.5 | ± | 3.2* | 14 | ± | 15* |
|  | rep30 | 10.7 | ± | 3.1* | 29 | ± | 22* |
|  | rep60 | 10.6 | ± | 2.6* | 30 | ± | 22* |
| plasma dialysate before RIC with aspirin-pretreatment (n=18) | baseline | 14.7 | ± | 1.8 | 107 | ± | 21 |
|  | infusion | 16.6 | ± | 2.8* | 93 | ± | 23*† |
|  | washout | 16.5 | ± | 2.2* | 99 | ± | 22 |
|  | isch5 | 0.0 | ± | 0.0* | 1 | ± | 0* |
|  | isch25 | 0.0 | ± | 0.0* | 2 | ± | 3* |
|  | rep10 | 10.0 | ± | 3.3* | 18 | ± | 14* |
|  | rep30 | 10.3 | ± | 3.0* | 38 | ± | 21*# |
|  | rep60 | 10.0 | ± | 2.1* | 34 | ± | 15* |
| plasma dialysate after RIC with aspirin-pretreatment (n=18) | baseline | 14.8 | ± | 1.8 | 107 | ± | 13 |
|  | infusion | 16.7 | ± | 2.7* | 92 | ± | 15* |
|  | washout | 16.7 | ± | 2.5* | 96 | ± | 14 |
|  | isch5 | 0.0 | ± | 0.0* | 1 | ± | 1* |
|  | isch25 | 0.0 | ± | 0.0* | 3 | ± | 1* |
|  | rep10 | 9.6 | ± | 3.1* | 15 | ± | 9* |
|  | rep30 | 10.0 | ± | 2.4* | 32 | ± | 17* |
|  | rep60 | 10.0 | ± | 1.9* | 37 | ± | 15* |
| plasma dialysate before RIC with ticagrelor-pretreatment (n=18) | baseline | 15.4 | ± | 1.9 | 110 | ± | 22 |
|  | infusion | 16.9 | ± | 2.2* | 94 | ± | 18* |
|  | washout | 16.7 | ± | 2.0* | 100 | ± | 20 |
|  | isch5 | 0.0 | ± | 0.0* | 3 | ± | 1* |
|  | isch25 | 0.0 | ± | 0.0* | 5 | ± | 3* |
|  | rep10 | 10.6 | ± | 2.8* | 19 | ± | 14* |
|  | rep30 | 11.7 | ± | 3.0* | 32 | ± | 22*# |
|  | rep60 | 11.0 | ± | 2.4* | 43 | ± | 18*†‡ |
| plasma dialysate after RIC with ticagrelor-pretreatment (n=18) | baseline | 15.2 | ± | 1.3 | 108 | ± | 19 |
|  | infusion | 16.6 | ± | 2.0* | 94 | ± | 23*† |
|  | washout | 16.6 | ± | 1.8* | 99 | ± | 21 |
|  | isch5 | 0.0 | ± | 0.0* | 2 | ± | 0* |
|  | isch25 | 0.0 | ± | 0.0* | 4 | ± | 3* |
|  | rep10 | 9.7 | ± | 2.3* | 16 | ± | 13* |
|  | rep30 | 11.0 | ± | 2.4* | 36 | ± | 20*# |
|  | rep60 | 10.5 | ± | 1.8* | 40 | ± | 16*† |

Data are means±standard deviations. n: number of rats; baseline: last min of stabilization period before ischemia/reperfusion; CF: coronary flow; I/R: ischemia/reperfusion; infusion: first min of infusion with saline solutions, washed platelets, or plasma-dialysates; LVDP: left ventricular developed pressure; isch5/25: 5/25 min of ischemia; RIC: remote ischemic conditioning; rep 10/30/60: 10/30/60 min reperfusion; baseline CF and LVDP at baseline were compared between all hearts by one-way ANOVA; time courses for CF and LVDP of hearts infused either with saline solution, with washed platelets, or with plasma-dialysates were analyzed by two-way ANOVA for repeated measures and Fisher’s least significant difference post-hoc tests, respectively; *p<0.001 vs. baseline, respectively; ^α^p<0.001 vs. saline, saline+aspirin and aspirin+ticagrelor; #p<0.05 vs. before RIC without pretreatment; †p<0.05 vs. after RIC without pretreatment; ‡<0.05 vs. before RIC with aspirin- pretreatment.

**Online Resources Supplementary Methods**

**Isolated buffer-perfused heart**

Hearts were excised and arrested in cold saline (8°C, supplemented with unfractionated heparin 300 IU/Ml). The aorta was immediately cannulated, and hearts were mounted on a Langendorff-apparatus and perfused with modified Krebs-Henseleit buffer (in mmol/L: NaCl 118.0, KCl 4.7, MgSO_4_ 16.0, KH_2_PO_4_ 1.2, glucose 5.6, NaHCO_3_ 24.9, sodium pyruvate 2.0, CaCl_2_ 2.0; gassed with 95% O2 and 5% CO2 in a pre-warmed reservoir, pH 7.40) at constant pressure of 65-70 mmHg. Coronary flow (CF) was measured with an inline ultrasonic flowprobe (TS410, Transsonic Systems Inc., Ithaca, NY, USA) above the aortic cannula. A fluid-filled latex balloon was inserted into the left ventricular (LV) cavity and connected to a pressure transducer (Codan-PVB, Lensahn, Germany) to measure LV pressure. End-diastolic LV pressure was set to 5-15 mmHg at baseline by graded balloon inflation during the initial 5 min perfusion. LV developed pressure (LVDP) was calculated as the difference between peak and end-diastolic LV pressure. CF, end-diastolic and peak LV pressure were continuously recorded (LabChart 8, AD Instruments Pty LTD, New South Wales, Australia). Hearts were allowed to stabilize for 10-20 min. Preparations with CF <10.0 mL/min or >18.5 min or LVDP <60 mmHg after the stabilization period were excluded. Twenty-four isolated heart preparations in total did not meet baseline criteria and were thus excluded from further analysis. Heart rate was kept constant at 360 beats per min by right atrial pacing. Hearts were immersed in pre-warmed oxygenated Krebs-Henseleit buffer. The temperatures of the perfusion and immersion buffers were monitored with probes in the aortic cannula and in the immersion buffer chamber throughout the experiment and kept between 37.5°C and 37.8°C by heat exchangers. Washed platelets were then infused into the aortic cannula using a low adhesive Teflon tubing at a flow rate which substituted 10% of the measured CF flow rate for 8 min, followed by a 2 min washout period. Plasma-dialysates were also infused for 8 min, followed by a 2 min washout period. Hearts were then subjected to 30 min/120 min global I/R. The infusion of plasma-dialysate corresponded to that used in our previous studies on the humoral transfer of remote ischemic conditioning's cardioprotection with similar experimental protocols ^1-5^. Dilution and total infused volume of washed platelet solutions were adapted to that of plasma-dialysate infusion. Perfusion pressure, temperature, pH, and oxygen saturation of the perfusion buffer were neither affected by infusion of washed platelets nor by infusion of plasma-dialysates. Ischemia and reperfusion (I/R) were induced by 30 min full stop of perfusion and subsequent 120 min reperfusion. CF and LVDP were calculated as mean values during the last min each of the stabilization period (baseline), at 5 and 25 min ischemia and at 10, 30 and 60 min reperfusion, respectively. The hearts were frozen in CryomatrixTM (Thermo Fisher Sientific, Schwerte, Germany) at -20°C and cut into transverse 2 mm thick slices. Infarcted tissue was demarcated by staining with 0.09 mol/L sodium phosphate buffer containing 1.5% triphenyl tetrazolium chloride (TTC) at 37°C for 5 min. Stained slices were photographed from both sides. The total slice area and the infarcted areas were quantified by computer-assisted planimetry (ImageJ 1.48v, National Institutes of Health, Bethesda, Maryland, USA), and infarct size was calculated as percent of the sum of left and right ventricular mass (% of ventricular mass).

1. Skyschally A, Gent S, Amanakis G, Schulte C, Kleinbongard P and Heusch G. Across-species transfer of protection by remote ischemic preconditioning with species-specific myocardial signal transduction by reperfusion injury salvage kinase and survival activating factor enhancement pathways. *Circ Res*. 2015;117:279-288.

2. Hildebrandt HA, Kreienkamp V, Gent S, Kahlert P, Heusch G and Kleinbongard P. Kinetics and signal activation properties of circulating factor(s) from healthy volunteers undergoing remote ischemic pre-conditioning. *JACC Basic Transl Sci*. 2016;1:3-13.

3. Skyschally A, Kleinbongard P, Lieder HR, Gedik N, Stoian L, Amanakis G, Elbers E and Heusch G. Humoral transfer and intra-myocardial signal transduction of protection by remote ischemic perconditioning in pigs, rats, and mice. *Am J Physiol Heart Circ Physiol*. 2018;315:H159-H172.

4. Lieder HR, Kleinbongard P, Skyschally A, Hagelschuer H, Chilian WM and Heusch G. Vago-splenic axis in signal transduction of remote ischemic preconditioning in pigs and rats. *Circ Res*. 2018;123:1152-1163.

5. Lieder HR, Skyschally A, Heusch G and Kleinbongard P. Plasma from remotely conditioned pigs reduces infarct size when given before or after ischemia to isolated perfused rat hearts. *Pflügers Arch*. 2019;471:1371-1379.
